# Translating clinical and patient-reported data to tailored shared decision reports with predictive analytics for knee and hip arthritis

**DOI:** 10.1007/s11136-020-02557-8

**Published:** 2020-06-19

**Authors:** Patricia D. Franklin, Hua Zheng, Christina Bond, Danielle C. Lavallee

**Affiliations:** 1 Department of Medical Social Sciences, Northwestern University Feinberg School of Medicine, 625 North Michigan Ave, Room 21-051, Chicago, IL 60611 USA; 2 University of Massachusetts Medical School, Worcester, MA USA; 3 Patient Research Group, LLC, Syracuse, NY USA; 4 University of Washington School of Medicine, Seattle, WA USA

**Keywords:** Patient-reported outcomes, Osteoarthritis, Predictive analytics, Shared decision-making, Total joint replacement

## Abstract

**Introduction:**

New informatics tools can transform evidence-based information to *individualized* predictive reports to serve shared decisions in clinic. We developed a web-based system to collect patient-reported outcomes (PROs) and medical risk factors and to compare responses to national registry data. The system generates predicted outcomes for individual patients and a report for use in clinic to support decisions. We present the report development, presentation, and early experience implementing this PRO-based, shared decision report for knee and hip arthritis patients seeking orthopedic evaluation.

**Methods:**

Iterative patient and clinician interviews defined report content and visual display. The web-system supports: (a) collection of PROs and risk data at home or in office, (b) automated statistical processing of responses compared to national data, (c) *individualized* estimates of likely pain relief and functional gain if surgery is elected, and (d) graphical reports to support shared decisions. The system was implemented at 12 sites with 26 surgeons in an ongoing cluster randomized trial.

**Results:**

Clinicians and patients recommended that pain and function as well as clinical risk factors (e.g., BMI, smoking) be presented to frame the discussion. Color and graphics support patient understanding. To date, 7891 patients completed the assessment before the visit and 56% consented to study participation. Reports were generated for 98% of patients and 68% of patients recalled reviewing the report with their surgeon.

**Conclusions:**

Informatics solutions can generate timely, tailored office reports including PROs and predictive analytics. Patients successfully complete the pre-visit PRO assessments and clinicians and patients value the report to support shared surgical decisions.

**Electronic supplementary material:**

The online version of this article (10.1007/s11136-020-02557-8) contains supplementary material, which is available to authorized users.

## Introduction

“Big data” and informatics tools have the potential to transform evidence-based information to *individualized* predictive reports to serve shared treatment decisions between clinicians and their patients. In the future, real-world evidence—defined as data collected outside the research environment from electronic health records (EHRs), registries, claims databases, and patients—will be transformed to improve and tailor shared decisions and to drive uniform excellence in care [1]. However, today’s EHRs are missing key information needed to provide patient-centered care, particularly among patients with knee and hip arthritis [2]. Included among the missing information are patient-reported health status and symptoms, such as pain and functional limitation, values and culture, and goals and care preferences [3]. To optimize real-world evidence, patient-generated data, including patient-reported outcomes (PROs), are increasingly collected in EHRs to inform knee and hip arthritis decisions. Real-time access to big data during the clinician-patient encounter provides an opportunity to make the best treatment decisions in individual situations [4]. These aggregate datasets can be transformed to *individualized* information to guide shared decision-making between patients and clinicians in arthritis care [5]. Efforts to transform comprehensive real-world evidence to individualized, actionable information require health systems to develop best practices for integrating PROs with real-world data to support shared decision-making [6].

Arthritis care through Shared Knowledge (A.S.K.) is a PCORI-funded study that developed and is evaluating evidence-based shared decision-making, including PRO measures that have become standard of care in knee and hip arthritis clinics [7]. The A.S.K. study system transforms data collected in the national Function and Outcomes Research for Comparative Effectiveness in Total Joint Replacement (FORCE-TJR) registry to individualized information for shared decision-making between orthopedic clinicians and patients newly evaluated for knee or hip arthroplasty [8, 9]. The translation of FORCE-TJR’s PRO data to real-time shared decision tools serves as proof of concept for both the complementary interaction between national databases and EHRs and the future value of PROs in predictive analytics to guide patient decisions.

To evaluate the effectiveness of tailored PRO-based shared decision reports, the A.S.K. study developed a decision tool for patients with advanced knee and hip osteoarthritis considering treatment options, including total joint replacement (TJR). Second, to collect the data needed for the A.S.K. report, and generate the graphical report, the study built a web-based informatics platform that regularly integrates updated national registry data with point-of-care information. Last, the study defined the implementation procedures required to collect data and prepare the A.S.K. report for use at the time of patient visit to busy orthopedic clinics. This paper summarizes the report development process, web-based system to support data capture and reporting, and the office procedures for implementing the report.

## Methods

### Designing the web-based data collection and reporting system to generate tailored reports

**Fig. 1:**
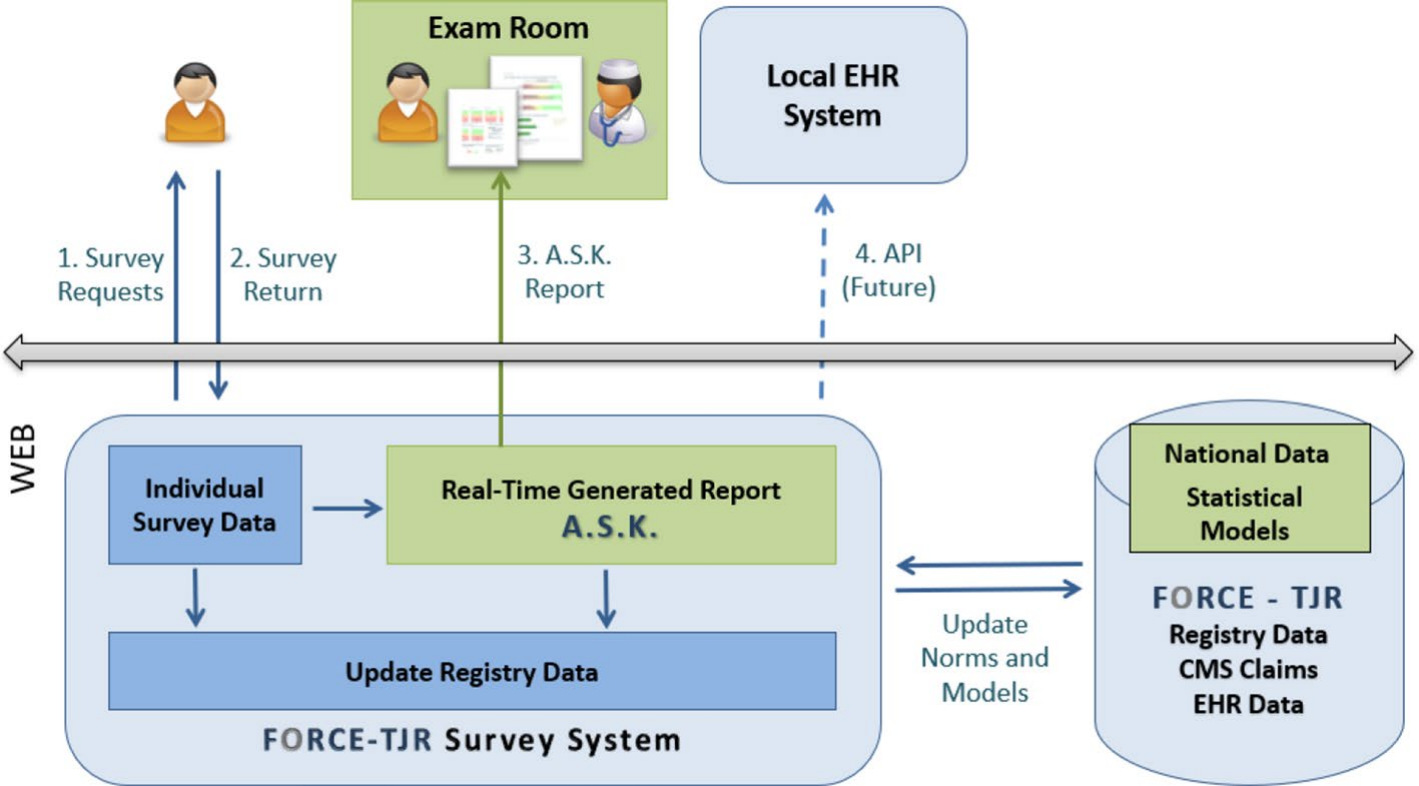
A.S.K. web-based system to generate individualized reports to support shared decision-making

The A.S.K. web-based informatics system includes the following functions in order to generate a tailored report at the point of clinical decision-making: (1) email delivery of unique web links to collect PRO and patient-reported clinical risk data (e.g., smoking, BMI, depressive symptoms) from patients at home or in office prior to clinician visit; (2) automated statistical processing of patient responses by comparing each individual to the FORCE-TJR national registry of 35,000 TJR patients with parallel demographic, comorbidity, and PRO data; and (3) computation of *individualized* estimates of likely pain relief and functional gain after surgery. The A.S.K. system then generates a real-time report with (a) the individual’s pain and function scores compared to national data, (b) key comorbid risk factors, (c) a decision grid for non-surgical options, and (d) individualized estimates of likely pain relief and functional gain based on the experience of similar patients to support shared decision-making between the patient and clinician at the office visit. Finally, (4) patient data are returned through batch feeds to the local sites’ EHR systems. Figure 1 illustrates the steps to produce the A.S.K. report for office use and to store data both in the local EHR and the national registry.

### Defining the components and presentation of the A.S.K. report

Increasing numbers of arthroplasty clinical practices use PROs to measure the severity of knee and hip osteoarthritis patients’ pain and function [10, 11]. Prior research demonstrates that PROs, combined with demographic and clinical risk factors, can explain statistical variation in 30 day readmissions and in pain and function one year after TJR [12–14]. Thus, the A.S.K. report wanted to display pain and function sub-scores of the Knee and Hip Osteoarthritis Outcome Scores (KOOS, HOOS) [15, 16] as well as patient risk factors that research associates with post-TJR symptom improvement or complications. Predictive analytics could then use these data to generate likely TJR outcomes based on patients with similar clinical symptoms and PROs captured within the national FORCE-TJR registry.

Our prior analyses of national data with outcomes at 12 months after TJR surgery documented wide variation in pain and function and the ability to predict discrete sub-groups of greater and lesser post-operative improvement [13]. These initial nomothetic models and subsequent FORCE-TJR analyses support that, in general, key pre-operative patient symptom and clinical variables are associated with greater or lesser pain relief and functional gain after surgery [17–19]. In addition, preliminary multivariable models developed in preparation for the A.S.K. study identified that *pre-TKR* attributes are significantly (*p* < 0.05) associated with specific activities, such as a greater likelihood of pain-free walking at 12 months after surgery: not obese, no knee pain at rest, no pain walking, excellent or very good general health, not feeling down-hearted or blue, and no history of prior ipsilateral knee procedure. Similarly, models combining pre-surgical comorbidity measures and patient-reported symptoms could predict readmission or not with good discrimination (*C* = 0.79 (knees); 0.86 (hips)) [12]. Using parallel pre-surgical patient-reported and clinical variables, models predicting likely 12 month post-TJR joint pain, joint-specific function, and global function were developed for the A.S.K. report.

To define the content, sequence, and visual presentation of these data in the A.S.K. report, iterative clinician key informant interviews were conducted. Five experienced arthroplasty surgeons from MA, CT, NY, and PA, a nurse practitioner, and a physical therapist participated in interviews. Clinicians reviewed literature summarizing the association between key patient and clinical factors and surgical outcomes, including pain relief, functional gain, and complications. Interviews began with open-ended questions as clinicians examined different graphical representations of the data. Second, the study’s patient advisers reviewed draft reports. Interviewers probed to validate key domains to include in the A.S.K. report. The interviewer captured initial impressions and observations in “quick notes”. The interview transcripts were analyzed using standard qualitative analysis techniques [20, 21] and compared with results from other studies examining the use of educational aids in orthopedics**.** Grounded theory coding techniques were employed [22] focusing on the underlying processes of TJR decision-making. Transcripts for each interview were reviewed and themes and categories of like responses identified (e.g., by question topic, by positive/negative). Multiple drafts of the A.S.K. report templates with color-coded symptom severity were reviewed with the clinicians to refine specifications. In addition, a draft decision grid with common non-surgical treatment options based on professional society recommendations was reviewed [23, 24].

**Fig. 2:**
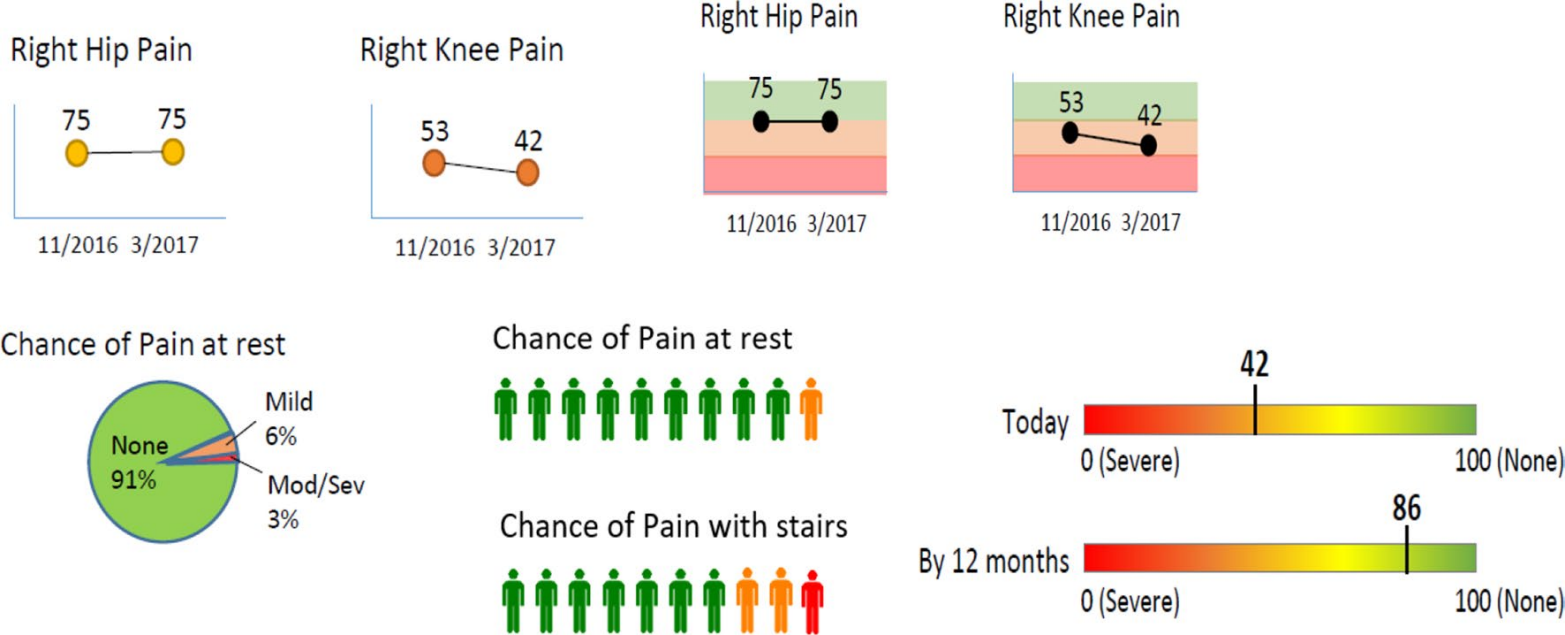
Draft options for data display in the A.S.K. report

To further refine the A.S.K. report presentation for clarity and interpretation, patients were recruited from orthopedic centers of participating surgeon advisors, with attention to diverse races and ethnicities. To anticipate the needs of future report users, adults (n = 16) who had recently made decisions about undergoing TJR surgery were identified to guide refinement of report presentation, priority outcomes, and information for non-operative treatments. Test patients included both adults who decided for and against elective TJR surgery to parallel future users of the report. The mean age of participants was 69.7 years (range 66–83 years); 56% male; 22% Hispanic and 22% Black; 22% had not elected TJR at the time of the interview. Semi-structured, in-person interviews were conducted by a single experienced clinician-qualitative researcher and were digitally recorded. In each interview, patients reviewed multiple A.S.K. report templates with optional data displays and were invited to describe their interpretation, provide feedback, and make suggestions to improve clarity. (See Fig. 2). Exploratory questions addressed: (1) ease of use of the tool; (2) comprehension of the tool content; (3) most useful elements, and most- or least-recalled elements; (4) relevance of the outcome data; and (5) general suggestions for improving the tool. Draft reports included varying visual data presentations such as bar graphs, pie graphs, word clouds, and people icons to represent proportions. Color coding with red representing the most severe symptoms and green the healthiest were applied. Reports were revised between interviews, based on sequential feedback. Consensus on report presentation was reached after nine 60- to 90-min interviews.

Using a site-level, cluster randomized design, the A.S.K. study will evaluate if this PRO-based shared decision tool enhances patients’ decision quality and/or influences treatment choices and outcomes. Because osteoarthritis is the most common disabling condition in the US and TJR is the costliest inpatient procedure, the decision about use of TJR is an important proof of concept [25]. Lessons learned from this study can inform future methods to translate large databases to individualized shared decision reports with predictive analytics.

## Results

### A.S.K. report design and presentation

Clinicians advised that both patient-reported symptoms (e.g., pain, limitations in function) and modifiable clinical risk factors important to predicting outcomes (e.g., BMI, smoking, emotional health) be presented simultaneously to frame both disease severity and surgical risks in the discussion. Because osteoarthritis pain can affect multiple joints, surgeons preferred that separate pain graphs be presented for right and left knees and hips. To align with emerging orthopedic norms and CMS-endorsed measures, surgeons recommended that the report first display knee and hip-specific pain scores (KOOS and HOOS, respectively) and then graph joint-specific function (KOOS or HOOS ADL) and global physical function [23, 24]. The clinical risk factors displayed are variables that are independently significant in prediction models of likely outcomes and are supported by prior research described above. Specifically, both young and old ages, diabetes [26], smoking, very high or very low BMI [27], advanced co-morbidities per Charlson index [13], co-existing pain in the non-operative knees and hips, and moderate to severe low back pain [28] have all been associated with higher complications and poorer TJR outcomes.

Clinicians endorsed the presentation of the calculated predicted likely benefits of surgery (e.g., pain relief and functional outcomes). However, because serious adverse event rates are small, and prediction models less precise, they suggested that national hospital readmission rate ranges be described [24]. Last, clinicians advised that national professional society best practice guidelines serve as the source of information for the decision grid [23]. At the conclusion of the clinician interviews, refined draft reports were developed for patients to guide the final data presentation and content.

The majority of patients—both advisors and in clinic—preferred simple bar graphs to other data displays, including pie charts and people icons. Participants preferred consistent backgrounds of red (severe osteoarthritis), yellow, and green (healthy) for patient-reported outcome trended reports because it placed the number or percentage in context across each report component. The addition of an arrow pointing from advanced symptoms to healthy symptoms aided interpretation. In addition to predicted likely post-operative pain and function *scores*, patients wanted to add the likelihood of being pain-free with three key activities, pain at rest (or sleeping) or when walking or climbing stairs.

Online Appendix 1 includes the final three-page A.S.K. report. Page 1 displays pain scores for both right and left knees and hips as well as function scores. In addition, medical and musculoskeletal risk factors associated with post-TJR outcomes are presented. Page 2 includes the predicted analytics for likely one-year post-TJR pain, function, and likelihood of being pain-free with key tasks. Of note, both arthritis-specific and generic function measures are displayed as the surgery is expected to improve joint-related function, but generic physical function, by definition, is affected by co-existing health conditions and may or may not improve. Page 3 includes a decision grid with comparisons of common non-operative treatments including medications, knee injections, and physical therapy. Patients defined the questions to include such as potential impact, length of treatment time, and need to repeat the treatment.

Both patient and clinical participants believed the content of the report was important to making a decision regarding elective TJR surgery. Participants stated that a shared decision report with individualized PROs assisted them in understanding the severity of their osteoarthritis, and informed decisions regarding elective TJR surgery. Beyond the report, a subset of participants believed that speaking with other patients who had previously made the decision for or against TJR surgery would be helpful.

The lead of the patient advisory group summarized the group’s sentiments:

In my mind [the survey] is part of the treatment plan. A lot of times they leave the patient out. They give the diagnosis and tell them to take that pill or whatever. By giving the answers to the questions, it helps you be involved in the actual decision-making and to understand why.

### Deploying the A.S.K. report in clinical care

With PCORI funding, the A.S.K. web-based data collection and reporting system was implemented at 12 orthopedic offices with 26 participating surgeons in an ongoing site-level, cluster randomized trial. The University of Massachusetts Medical School Institutional Review Board for Human Subjects serves as the single IRB of record for all sites. In addition to IRB reviews, information system security reviews were completed at all participating sites prior to study initiation. Each site approved the A.S.K. system as meeting optimal standards for privacy and data security standards. Finally, prior to implementation, the 26 site surgeons, mid-level practitioners, and clinic staff were educated on the use of the report, the value of shared decision-making, and each of their roles in the process of data capture and report generation. Staff sessions were site specific and tailored to the local office flow.

To date, 7891 patients completed the PRO and risk assessment before the office visit. Of these, 73% had a diagnosis of knee or hip osteoarthritis making them eligible for the study and 56% of eligible patients (*n* = 3157) consented to complete a post-visit structured interview. Refusals to participate were uncommon (12%); “unable to reach by phone” after the office visit was the most common reason that patients were not consented. Consenting patients have a mean age of 65.7 years, 61% female, mean BMI of 32.0. See Table 1 for demographics and risk factors at time of office visit.

**Table 1:** Demographics and clinical descriptors of patients with osteoarthritis who used A.S.K. report

	Knee	Hip	Total
	Mean/SD	Mean/SD	Mean/SD
Age	65.5/9.9	64.7/10.3	65.2/10.0
BMI	32.8/6.8	30.5/6.2	32.0/6.7
MCS	55.2/11.9	54.5/11.9	54.9/11.9
PCS	30.5/9.5	30.2/9.6	30.4/9.5
Pain (KOOS or HOOS)	42.0/16.6	40.6/17.8	41.5/17.0
ADL (KOOS or HOOS)	47.5/20.1	47.3/20.8	47.4/20.3

	%	%	%
Charlson Index			
0	58.1	60.8	59.0
1	21.1	17.4	19.8
2, 3, 4, 5	12.4	11.2	12.0
> 5	8.5	10.6	9.2
Smoker			
No	90.9	88.1	90.0
Yes	9.1	12.0	10.1
Low back pain			
None/mild	73.8	65.6	71.0
Mod/severe	26.2	34.4	29.0

All data are from the initial visit

Offices received dedicated color printers to assure that the patient and clinician received color-coded reports for shared review during the office visit. Overall, A.S.K. reports were printed for 93% of patients with completed data. At 2 weeks post-visit, 68% recalled reviewing the report with their surgeon (range 45–81%) and a majority of patients valued the information. In addition, 86% of patients at the 6 month post-visit time point and 87% at the 12 month time point completed a repeat PRO assessment of their arthritis symptoms.

Web-based tracking reports allow sites to monitor data capture and report rates. Consented patients complete a post-visit structured interview to assess report use and decision quality. Study enrollment, shared decision report evaluation, and 6 and 12 month symptom monitoring are ongoing. The impact of components of the A.S.K. report on shared decision quality and outcomes will be analyzed according to the milestones described in the research protocol.

## Discussion

This paper summarizes the design and implementation of a web-based informatics system that collects pre-visit PRO measures and medical risk factors, and integrates these data with nationally representative data to generate an evidence-based shared decision report to guide patients and clinicians discussing TJR surgery as a treatment option. The web-based system components allow real-time individualized reports. Finally, office implementation procedures and report use are presented.

Uniquely, the A.S.K. report transforms data captured in a registry to an individualized report available at the point of care. The FORCE-TJR national registry collects pre-specified patient and clinical data at pre-determined, clinically relevant intervals *across health delivery settings*, rather than from a single health system or EHR. Similarly, national registries in the United Kingdom, Sweden, and Australia follow patients over time and collect annual PROs [10]. Thus, registry data include uniformly defined metrics from a national sample of patients. In contrast to a registry, EHR data are documented in inconsistent formats, at inconsistent time intervals, and are limited to visits within each discrete health system. For optimal use of real-world data in predictive analytics and clinical decision-making, the data must be complete, capture longitudinal outcomes, and include unbiased samples. Because the FORCE-TJR registry has complete and nationally representative data, it serves this database function well.

Beyond collecting consistent, comprehensive data, the A.S.K. system can execute advanced statistics and generate detailed visual reports. Centralized statistical processing and visualization functions are efficient to manage future model refinements, such as adding additional languages or visual presentations to address the diversity of numeracy and literacy of future users. In addition to the individual patient reports, participating surgeons and sites receive aggregate patient profiles to compare their patients to national populations to better understand the profile of referred patients.

**Fig. 3:**
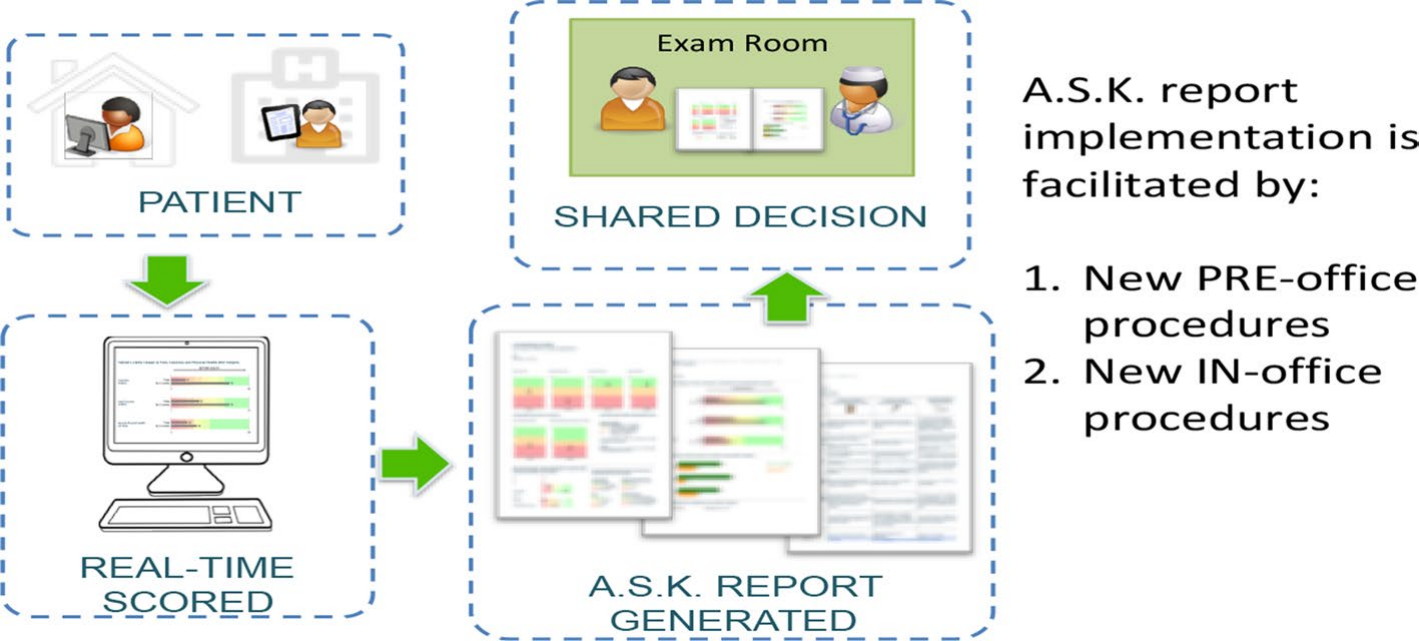
Pre-visit procedures to assure A.S.K. reports are available for shared decision support between new patients and clinicians

A.S.K. clinical site implementation is addressing logistical challenges such as defining new office procedures to identify upcoming patients presenting for their first visit to the office. While most EHR systems can flag a new patient to the office for administrative reasons, the pre-visit information may not include updated email addresses to facilitate delivery of the secure link prior to the visit. To facilitate completion, study staff call patients to verify contact information and facilitate survey completion in advance of the visit. The surgeon’s office mails a paper letter simultaneously to introduce the study staff. The A.S.K. email includes a tailored note from the surgeon requesting that the patients complete the assessment prior to the office visit. In addition, the unique link embedded in the email enables patients to directly complete the assessment without the additional step of establishing a username and password. For patients who do not complete the A.S.K. assessment in advance of the office visit, tablets are available at the office to complete the survey and generate the report. However, busy offices are inconsistent in their collection of these data in the waiting room. As predicted by patient interviewees, patients reported that the A.S.K. system is easy to use (see Fig. 3).

Lessons learned while implementing the A.S.K. report in busy ambulatory surgeon offices can inform broader efforts to successfully capture and integrate PROs and other patient-generated data in daily care decisions. Today’s EHRs effectively capture and manage clinical data and visit documentation. However, patient-reported data capture chronic symptom trajectories between visits and are important to shared decisions about chronic conditions [14]. New A.S.K. virtual, email, and phone procedures collect pre-visit patient data and longitudinal outcomes, enabling integration of both patient and clinical data to support treatment decisions.

The A.S.K. shared decision report is a novel decision aid with individualized information designed by patients and clinicians. The A.S.K. study has enrolled over 3100 patients to date and patients at one-half of the sites receive the three-page report with the predictive analytics. An additional 3000 patients will be recruited for a total of 6000 patients of 36 surgeons in 11 states. Study implementation across a large volume of patients from diverse geographic regions will allow the research team to further refine the report, as needed, based on feedback. When enrollment is complete, the investigators will conduct both qualitative and quantitative evaluations of patient and clinician use of the report. All participating patients complete a structured decision quality interview after their visit to assess the value of the predictive analytics and evidence-based comparative information about alternative treatments. Clinicians and a sample of patients will be interviewed to further characterize both the strengths and weaknesses of the report.

At the conclusion of this research, if the A.S.K. reports provide incremental value to patient decision-making, future application programming interfaces can be developed to return individual A.S.K. reports to individual EHR files. EHR modifications will require the ability to match patient identifiers between the A.S.K. system and the local record, to create new fields to store A.S.K. survey variables and estimates, and to support automated and secure data transfer interfaces.

Future learning health systems will require information technology to rapidly integrate evidence of national best practice with individual patient data captured at the time of care [29]. Lessons from the A.S.K. shared decision report design, implementation, and evaluation has the potential to enhance patients’ decision quality and influence treatment choices and outcomes. The A.S.K. prototype is evaluating one implementation model to shape future systems that inform shared decision-making with real-world evidence, including patient-reported data.

## Electronic supplementary material

Below is the link to the electronic supplementary material.

Supplementary file1 (DOCX 17 kb)

Supplementary file2 (PDF 293 kb)
