# Supplementary material for: Translating clinical and patient-reported data to tailored shared decision reports with predictive analytics for knee and hip arthritis
Source: Qual Life Res. 2020 Jun 19;30(11):3171–8. doi: 10.1007/s11136-020-02557-8 (PMC8528740; doi:10.1007/s11136-020-02557-8)
Supplement: Supplementary file 1 — Supplementary file1 (DOCX 17 kb) [file 11136_2020_2557_MOESM1_ESM.docx]

**Appendix**

Appendix 1. A.S.K. 3-page report with descriptive PROs and clinical risk factors (page 1), likely predicted TJR outcomes (page 2), and decision grid of non-operative treatments (page 3).
